# Unraveling the interparticle strain dynamics in battery electrodes

**DOI:** 10.1093/nsr/nwag059

**Published:** 2026-01-28

**Authors:** Xikun Zhang, Hongtao Qu, Bao-Lian Su

**Affiliations:** State Key Laboratory of Advanced Technology for Materials Synthesis and Processing, Wuhan University of Technology, China; Laboratory of Inorganic Materials Chemistry (CMI), University of Namur, Belgium; Laboratory of Inorganic Materials Chemistry (CMI), University of Namur, Belgium; State Key Laboratory of Advanced Technology for Materials Synthesis and Processing, Wuhan University of Technology, China; Laboratory of Inorganic Materials Chemistry (CMI), University of Namur, Belgium; Clare Hall, University of Cambridge, UK

Intercalation-based lithium-ion batteries (LIBs) have fundamentally transformed modern energy storage technologies [1,2]. However, performance degradation caused by chemomechanical effects like electrode swelling and crack formation remains a major obstacle [3]. While previous studies have focused on static structural defects, the dynamic evolution of strain during electrochemical cycling remains insufficiently understood [4]. A groundbreaking study by Sun *et al.* published in *Science* addresses this gap [5]. By integrating multimodal *operando* microscopy and diffraction, Sun and co-workers revealed that real-time strain evolution in layered cathodes originates from particle-scale electrochemical heterogeneity and asynchronous chemomechanical coupling, reshaping our understanding of degradation mechanisms.

**Figure 1.**
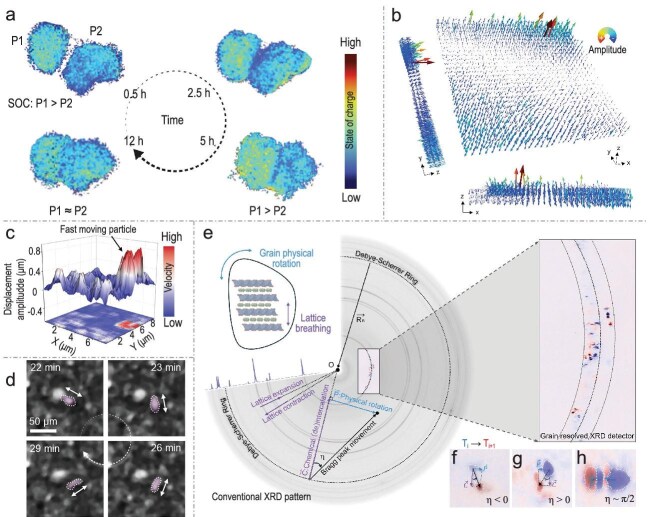
(a) TXM SOC mapping using Ni K-edge energy at 0.5, 2.5, 5 and 12 h (charging rate: ∼C/12). (b) Visualization of the electrode strain through deformation vector field extracted through optical flow analysis. (c) Amplitude of particle lateral displacement. (d) Localized particle rotation observed through *in situ* optical microscopy. (e) Schematic illustration of the synchrotron-based, grain-resolved XRD. (f–h) Selected Bragg peaks with different chemomechanical behaviors upon cell cycling that show a different combination of chemical (de)intercalation and physical rotation. These maps are differentially fused using two sets of consecutively acquired patterns (T_i_, blue spots; T_i+_, red spots). The time span between two adjacent time steps is 10 min. Adapted with permission from Sun *et al*. [5].

Using a custom-designed capillary cell with a free-standing single-crystalline LiNi_0.6_Mn_0.2_Co_0.2_O_2_ (NMC) cathode, Sun and co-workers employed *operando* transmission X-ray microscopy (TXM) to track particle movements during slow charging. They observed significant particle rearrangements, with some grains moving toward each other and establishing physical contact over time. Correlating these motions with state of charge (SOC) maps from Ni K-edge energy shifts revealed persistent interparticle SOC heterogeneity during early charging (charging rate: ∼C/12; Fig. 1a). Crucially, once particles made physical contact, their SOCs converged, demonstrating interparticle charge transfer and redox coupling. This early-stage strain accumulation is a system-level response to relieve stresses from asynchronous SOC evolution among particles. Such particle clustering and local charge redistribution can increase inter-cluster impedance, redirect local currents, and cause uneven active material utilization, which are critical contributors to performance fade.

To bridge model systems with practical batteries, Sun and co-workers performed 3D *in situ* X-ray laminography on a compressed single-layer pouch cell with an Li- and Mn-rich (LMR) polycrystalline cathode. Non-uniform particle movements with distinct in-plane and out-of-plane displacements led to electrode wrinkling. Most electrode strain accumulated at 4.4 V [6], prior to the onset of oxygen redox reactions (typically linked to gas release and stress), indicating that electrode-level strain initiates early in the charge cycle and is independent of oxygen activity [7,8]. The strain pattern combined slow-varying curvature with high-frequency wrinkling with pronounced out-of-plane deformation at the edges (Fig. 1b). Smaller particles exhibited greater out-of-plane displacement, likely due to faster (de)lithiation kinetics. These findings confirm electrode strain is a universal, early-emerging phenomenon in practical cells, including commercial cylindrical and pouch formats.

In both cell types, Sun and co-workers observed that generally slow, progressive motion was interspersed with sporadic, rapid particle movements, where some particles reached speeds of tens of micrometers per minute. These sparse, random events were analyzed using *operando* optical microscopy and an event-triggered machine learning model (Fig. 1c and d). The analysis confirmed that such motions involve rapid rotation and translation, likely caused by particles detaching from the electrode matrix due to abrupt local current changes. These transient dynamics introduce localized mechanical perturbations that may exacerbate microstructural instability.

To probe fundamental strain-generation mechanisms, Sun and co-workers conducted grain-resolved synchrotron X-ray diffraction (XRD; Fig. 1e). By tracking Bragg peak movements on a Debye–Scherrer ring, they monitored individual grain responses, with radial shifts corresponding to chemical (de)intercalation ($\mathop C\limits^{\rightarrow} $) and tangential shifts reflecting physical grain rotation ($\mathop P\limits^{\rightarrow} $; Fig. 1f–h). The analysis of hundreds of grains revealed a spectrum of behaviors, including coupled $\mathop C\limits^{\rightarrow} $-$\mathop P\limits^{\rightarrow} $ motion, $\mathop P\limits^{\rightarrow} $-dominated motion (rotation without significant intercalation) and motion limited to $\mathop C\limits^{\rightarrow} $ (intercalation without rotation). A significant proportion displayed $\mathop P\limits^{\rightarrow} $-dominated motion, indicating that even electrochemically inactive grains participate in strain transmission through mechanical interactions.

This led to a proposed hierarchical strain cascade mechanism. Chemically active grains with coupled $\mathop C\limits^{\rightarrow} $-$\mathop P\limits^{\rightarrow} $ dynamics act as primary strain generators. Their anisotropic expansion drives intragranular rearrangements and transmits stress to neighboring grains. Inactive grains, in turn, undergo rotation-dominated motion, propagating strain across the electrode. This interplay creates a stress transmission network that converts localized electrochemical strain into mesoscale morphological changes. The decoupling of chemical and physical processes underscores the complexity of electrode chemomechanics.

Complementing experiments, Sun and co-workers developed an electro-chemomechanical model simulating strain evolution under electrochemical stimuli. Incorporating reaction heterogeneity, concentration polarization and stress accumulation, the model provides a theoretical framework for the observed dynamics. It emphasizes that electrode durability depends not only on mitigating chemical expansion but also on engineering microstructures that harmonize chemomechanical coupling in active regions while dissipating stress in inactive areas.

This study fundamentally advances the understanding of dynamic strain evolution in battery electrodes. It demonstrates that electrochemical heterogeneity at the particle scale drives mechanical stress through chemomechanical interactions, propagating across the electrode. The findings shift the focus from material-level optimization to electrode architectural design. To enhance battery longevity, future electrodes should incorporate adaptive, stress-resilient features, such as tunable elasticity, engineered porosity and regulated stack pressure. By linking particle-scale electrochemistry to electrode-scale degradation, the work highlights that durable batteries require tailored microstructural engineering. This represents a critical step toward designing next-generation, high-energy-density devices with improved mechanical durability.
